# Natural Antimicrobials in Dairy Products: Benefits, Challenges, and Future Trends

**DOI:** 10.3390/antibiotics13050415

**Published:** 2024-05-01

**Authors:** Maria Eduarda Marques Soutelino, Adriana Cristina de Oliveira Silva, Ramon da Silva Rocha

**Affiliations:** 1 Department of Food Technology (MTA), College of Veterinary, Fluminense Federal University (UFF), 24230-340 Niterói, Brazil; mariaems@id.uff.br (M.E.M.S.); adrianasilva@id.uff.br (A.C.d.O.S.); 2 Food Engineering Department (ZEA), College of Animal Science and Food Engineering (FZEA), University of São Paulo (USP), 13635-900 Pirassununga, Brazil

**Keywords:** foodborne diseases, natural antimicrobial compounds, bacteriocins, essential oils

## Abstract

This review delves into using natural antimicrobials in the dairy industry and examines various sources of these compounds, including microbial, plant, and animal sources. It discusses the mechanisms by which they inhibit microbial growth, for example, by binding to the cell wall’s precursor molecule of the target microorganism, consequently inhibiting its biosynthesis, and interfering in the molecule transport mechanism, leading to cell death. In general, they prove to be effective against the main pathogens and spoilage found in food, such as *Escherichia coli*, *Staphylococcus aureus*, *Bacillus* spp., *Salmonella* spp., mold, and yeast. Moreover, this review explores encapsulation technology as a promising approach for increasing the viability of natural antimicrobials against unfavorable conditions such as pH, temperature, and oxygen exposure. Finally, this review examines the benefits and challenges of using natural antimicrobials in dairy products. While natural antimicrobials offer several advantages, including improved safety, quality, and sensory properties of dairy products, it is crucial to be aware of the challenges associated with their use, such as potential allergenicity, regulatory requirements, and consumer perception. This review concludes by emphasizing the need for further research to identify and develop effective and safe natural antimicrobials for the dairy industry to ensure the quality and safety of dairy products for consumers.

## 1. Introduction

Despite the advances in health control measures in the food production industry [1], data from the World Health Organization (WHO) suggest that about 600 million people worldwide suffer from foodborne diseases (FADs), resulting in 420,000 deaths annually. Dairy products account for approximately 14% of these cases [2]. This is because dairy products can carry significant pathogens such as *Listeria monocytogenes*, *Salmonella* spp., *Staphylococcus aureus*, and *Escherichia coli* [3].

One of the biggest obstacles the food industry faces is preventing the growth of pathogenic and spoilage microorganisms, which, in addition to causing deterioration and off-flavor in dairy products, can generate food diseases worldwide [4]. As a result, the dairy sector has prioritized maintaining microbiological quality to ensure food safety and prevent economic losses. This has led to increased use of preservatives with antimicrobial properties in food formulations.

Considering the synthetic food conservators, despite a vast list, benzoates and sorbates are the most commonly used in food formulations because of their safe use history and low cost [5]. However, some studies suggest that these substances may have allergenic potential in sensitive individuals [6]. Furthermore, they can form toxic and carcinogenic by-products [7], which is a cause for concern among consumers. As a result, a growing demand for foods free from artificial preservatives puts pressure on the industry to focus on producing “clean label” products [8].

In this scenario, natural antimicrobial compounds are a promising alternative for conserving and increasing the shelf life of foods. They can be classified according to the source (microbial, plant, and animal), biological activity in organisms, and biosynthesis [9]. Bacteriocins are the most widely used antimicrobials from microbial sources, produced mainly by Gram-positive bacteria, including lactic acid bacteria (LAB) [10]. One example is nisin, which can be added directly to products in its purified form or produced in situ by probiotics, with efficacy described mainly in milk [11], yogurt [12], and cheeses [11,13]. Biopreservatives of animal origin have also been used in these products, such as lysozyme, lactoferrin, and chitosan [14,15,16,17]. These substances generally can directly inhibit microorganisms and biofilm formation and catalyze reactions that produce antimicrobial compounds [15,17,18]. In general, natural antimicrobials act in many ways, such as inhibiting biosynthesis and interfering with the transport mechanism of molecules, leading to cell death and other cell death stimulation [15].

Several studies have evaluated using plant extracts to replace conventional preservatives in dairy products, mainly cheese, yogurt, dairy beverages, and ice cream. This has resulted in an improvement in both the quality and shelf life of these products, as well as the enhancement of their sensorial aspects [19,20,21,22]. The use of essential oils (EOs) has also been studied in terms of inhibiting the growth of microorganisms within dairy matrices [23,24]. These studies have shown further benefits, such as improving the sensorial and nutritional aspects of formulations containing lactic acid [24]. Additionally, essential oils offer functional properties such as being anti-inflammatory, antioxidative, antispasmodic, and diuretic and having tonic action [25].

Although natural antimicrobials have been successfully used in the food industry, some factors intrinsic and extrinsic to the food can reduce their effectiveness [9,26]. Encapsulating these compounds has emerged as a promising technology to overcome this issue. It involves isolating bioactive substances in a protective coating, which improves their viability, stability, and solubility [27]. Studies have shown that capsules made from different materials can preserve biopreservatives and deliver them to the target location [27,28,29]. This approach can help maintain dairy products’ physicochemical and sensory properties [29].

The dairy industry and research centers are constantly exploring the potential of natural antimicrobials from different sources. In this regard, a review has been conducted to highlight the effects of applying such antimicrobials to dairy products. This review aims to provide a deeper understanding of the potential benefits and challenges of using natural antimicrobials in the dairy industry. This review also explores encapsulation as a promising alternative for increasing the viability of these compounds. This will help identify the most effective and safe natural antimicrobial options for dairy products, ensuring the quality and safety of the products for consumers.

## 2. Bacteriocins

Bacteriocins are antimicrobial peptides synthesized mainly by LAB, which can be added to food in purified or semi-purified form, or produced in situ by a starter culture [26]. These compounds can inhibit pathogenic and spoilage microorganisms, without altering the physical–chemical characteristics of the food, in addition to being easily digested by the human gastrointestinal tract [30]. However, the use of bacteriocins as food additives is still limited for several reasons, such as the low effectiveness in eliminating specific pathogens or the high cost of large-scale applications [31].

### 2.1. Nisin

Despite its limited effect on Gram-negative bacteria, molds, and yeasts, nisin is widely used in the dairy industry due to its strong action against important Gram-positive bacteria, such as *L. monocytogenes* and *S. aureus* [32]. Its antimicrobial activity is based on the mechanism of binding to the precursor molecule of the cell wall of the target microorganism, with consequent inhibition of its biosynthesis [33].

Felício et al. [34] evaluated the effects of this bacteriocin on the count of *S. aureus* in vitro and in situ in Minas Frescal cheese, observing that in vitro, nisin delayed the lag phase. On the other hand, in the in situ evaluation, the concentration of 500 UI∙mL^−1^ reduced the count of *S. aureus* in cheese and whey by 1.5 logarithmic cycles (*p* > 0.05). Meshref et al. [35] obtained similar results when studying the effect of nisin against *S. aureus* in Kareish cheese, in which the addition of 12.8 ppm nisin reduced the count from 4 × 10^8^ UFC/g to 1 × 10^2^ UFC/g after four weeks of storage.

Ning et al. [36] investigated the antibacterial activity of nisin synergistically with sucralose laurate (SL) in dairy beverages. Through alkaline phosphatase and peptidoglycan assays, fluorescence spectroscopy, and flow cytometry analysis, the authors observed that these associated compounds block the synthesis of peptidoglycan, damaging the integrity of the cell wall of *S. aureus* and inhibiting the activities of Na-ATPase in the cell membrane.

Nisin has variants A and Z, with nisin A being the most used in the industry as it is a safe and effective preservative when added within recommended limits. Oshima et al. [37] analyzed the effectiveness of nisin A in controlling *Bacillus thuringiensis*, *Bacillus cereus*, and *Paenibacillus jamilae* in a dairy-based dessert, demonstrating that 80 IU g^−1^ and 120 IU g^−1^ of this preservative were able to inhibit the growth of these bacteria in dessert puddings with 5% and 7.5% fat, respectively. Efficiency against spore-forming pathogens, such as Bacillus cereus, is a significant result as it can be aligned with the reduced need to expose the product to high temperatures for an extended period during heat treatment, thus ensuring better preservation of the product’s functional, nutritional, and sensorial characteristics.

Although the properties of nisin Z are still little studied, this variant has also demonstrated excellent preservative potential and high stability in dairy products [12]. Murat et al. [11] reported that strains of *Lactococcus lactis* subsp. lactis have high activity in the synthesis of nisin Z in fluid milk and cheeses, whose purified form was moderately inhibitory against Gram-negative bacteria and stable at temperatures between −20 °C and 4 °C for six weeks. Furthermore, nisin Z extended the shelf life of UHT milk at 30 °C by up to 6 weeks and was susceptible to pepsin (92%), lipase (89%), and catalase (84%) at 37 °C for 60 min.

Although the numerous advantages of using this bacteriocin have been proven, some factors may affect its effectiveness in dairy products [32]. For example, Modugno et al. [38] report that dairy products with a pH close to neutrality (pH > 6) can limit their efficiency due to undesirable structural variations, favoring β-folding. Van Tassel et al. [13] demonstrated that ferulic acid, either alone or in combination with nisin, can inhibit the growth of *Listeria innocua* and *L. monocytogenes* in fresh Hispanic cheese. Their study found that 4 mg/g of ferrulic acid was significantly more effective in inhibiting these bacteria than nisin Z. Fresh cheeses have a pH close to neutrality (about 6.0) and do not undergo maturation processes or the addition of LAB. Therefore, the use of nisin has limited effectiveness on these products. However, there is some contradictory evidence in the literature regarding the ideal pH value for nisin Z to be effective [11,13,38,39].

The interaction of nisin with compounds in the food matrix is another limiting factor of its application [12]. Wee et al. [12] characterized and quantified the degradation products of nisin A and Z by HPLC, suggesting that fruit-flavoring substances added to yogurt can promote the oxidation of nisin Z, reducing its effectiveness.

Furthermore, microbial resistance to nisin has already been described in cheese by Caprini et al. [39], who added nisin at concentrations of 100 or 500 IU∙mL^−1^ to Minas Frescal cheese but did not observe a reduction in *S. aureus* counts. The authors reinforce the theory that the resistance of certain microorganisms to nisin may vary geographically since different effects were observed in other regions [39]. However, this result may highlight the low effectiveness of nisin in products with a pH close to neutrality [13,38].

Although there is some promise, there is a lack of agreement on certain aspects essential to interpreting the data. For instance, some studies report the beneficial effects of fluid milk [11], while others report inefficiency at a pH close to neutrality [13,38,39]. Generally, interference with antimicrobial action may be more related to the composition of the matrix than to its pH. For instance, the action of nisin is limited due to its interaction with the casein micelle [40] and fat globules [41], which are present in a more concentrated form in cheese. Additionally, the presence of divalent cations that block the access of nisin to the membrane of pathogens is another limiting factor [42], and it is not discussed thoroughly in most articles.

### 2.2. Natamycin

Natamycin is a potent polyene–macrolide antifungal produced by strains of *Streptomyces natalenses* [43] and is recognized as safe (GRAS) by the Food and Drug Administration (FDA) in doses of up to 0.3 mg kg^−1^ [44]. It is generally used in the dairy industry to preserve cheeses as it presents excellent activity against surface-contaminating fungi and does not modify the sensorial characteristics of the product [43]. Several studies report the effectiveness of natamycin in inhibiting the growth of Penicillium spp. [45,46], *Aspergillus* spp. [47,48], and *Cladosporium* spp. [45,49], with most fungi and yeasts inhibited by 0.5–6 μg/mL and 1.0–5.0 μg/mL of this compound, respectively [50].

Research of natamycin application in dairy products is mainly limited to the conservation of cheese, whether its in situ addition or in the production of edible films and packaging [51,52]. In this scenario, the antifungal activity of natamycin (5, 10, and 20 ppm) and combined at 10 ppm with potassium sorbate (1%) was investigated by Ombarak and Shelaby et al. [51] in Tallaga cheese stored at 4 °C for 30 days, demonstrating inhibitory solid activity against fungi at all concentrations studied, including in synergism with potassium sorbate.

González-Forte, Amalv, and Bertola [45] demonstrated that natamycin in a cornstarch coating showed an inhibitory effect on the surface of semi-hard cheeses against fungi isolated from dairy factories, including *Penicillium* spp. In a similar study, Küçük et al. [52] developed alginate and zein films containing natamycin (100, 200, 500, 1000, 2000, and 4000 ppm) for Kosher cheeses, finding that the inhibitory activities of the coatings against *Aspergillus niger* and *Penicillium camemberti* increased as the natamycin concentration increased. Torrijos et al. [46] evaluated this compound’s in vitro antifungal activity against *Penicillium* spp. by using a spray treatment and in a hydroxyethylcellulose film on mozzarella. It was observed that concentrations between 1.6 and 3.1 μg/mL of natamycin promoted a reduction of 5.28 Log CFU/g and 4.15 Log CFU/g, respectively.

Finally, the antifungal activity of natamycin was also described in low-density polyethylene (LDPE) packaging by Anari et al. [53]. The modified LDPE with acrylic acid at different UV exposure times (0–10 min) was coated with natamycin, which covalently linked to the pendant functional groups, concluding that films treated for 6 min control the growth of *Rhodotorula mucilaginosa* and *Candida parapsilosis* and extend the shelf life of fermented milk by 23 days.

Natamycin is used in dairy factories in processes of immersion, surface application, or spraying. Despite not having many innovations, studies report its effectiveness against important molds and yeasts in foods. In this sense, the biggest trend is its use in packaging and active films, which can become a promising field for studies, especially when considering long-storage dairy products.

### 2.3. Reuterine

3-hydroxy-propionaldehyde (3-HPA) or reuterin is an antimicrobial naturally produced from glycerol by *Lactobacillus reuteri*, with inhibitory activity against a broad spectrum of Gram-positive and Gram-negative bacteria, protozoa, molds, and yeasts [54]. It is also recognized as Generally Recognized As Safe (GRAS) and Qualified Presumption of Safety (QPS) in the USA and Europe, respectively [55], being capable of inducing oxidative stress in microorganisms when reacting with thiol groups [56]. Several factors can influence the production of 3-HPA, as it directly depends on the conditions for growing L. reuteri, such as pH and temperature. Furthermore, the manufacturing conditions of the dairy product must be designed to favor its development.

*E. coli* is a common pathogen in cheeses, especially soft and fresh ones [57]. Severe cases of infection with this bacteria can lead to acute gastrointestinal conditions, in addition to kidney failure, hemolytic uremic syndrome (HUS), and death. Al-Nabuls et al. [58] investigated the survival of this pathogen in salty white cheeses with the addition of *Lactobacillus reuteri* at different temperatures (10 and 15 °C) and NaCl concentrations (10% and 15% *w*/*w*). The study revealed that *L. reuteri* in brine cheese at all tested concentrations significantly reduced *E. coli* O157:H7 counts after 28 days of storage. Similar results were described by Langa et al. [59], who evaluated the protective effect of this antimicrobial produced by *Lactobacillus reuteri* INIA P572 and 100 mM glycerol against *L. monocytogenes* and *E. coli* O157:H7 during the manufacture and ripening of semi-hard cheese, reporting the inhibition of the growth of both after 30 days.

The inhibitory activity of reuterin is also effective against contaminating fungi and was described in yogurts by Vimont et al. [54] and Pilote-Fortin et al. [60]. Vimont et al. [54] quantified the minimum inhibitory activity (MIC) and minimum fungicidal activity (MFC) of reuterin against a representative panel of contaminating fungi and yeasts in yogurt. As a result, there was a fungistatic and fungicidal effect, eliminating around 99.9% of the microorganisms tested at concentrations equal to or lower than 15.6 mM. Pilote-Fortin et al. [60] evaluated the stability and antimicrobial efficacy of this compound at 10 mM added to milk before processing stirred yogurt, finding total inhibitory activity against *Rhodotorula mucilaginosa* and *Mucor racemosus* in one week and reduction in *Aspergillus niger* after three logarithmic cycles within four weeks.

Although the results show efficiency against a wide group of microorganisms, the metrics for evaluating efficiency vary depending on the manufacturing methodology. LAB have the characteristic of using lactose as a substrate, the same used by some Gram-negtive bacteria, for example. In this sense, there is a more significant shortage of nutrients. This directly affects other microorganisms’ growth without knowing the real effect of reuterin against specific pathogens. However, its contribution is evident [58,59]. Using isolated reuterin is a more accurate alternative to verify its efficiency, presenting more significant results against microorganisms [60].

## 3. Antimicrobial Compounds from Animal Sources

Lysozyme and lactoferrin are important antimicrobial proteins found in milk and dairy products. They exhibit antibacterial and antioxidant properties and are effective against many bacteria. Several studies have shown that adding these proteins to dairy products can improve their microbial quality, sensory attributes, texture, and storage stability. They are also being evaluated for use in food packaging, and hydrolyzed versions may have greater antimicrobial activity and functionality.

### 3.1. Lysozyme

Lysozyme is an important antimicrobial found in animal sources, particularly in dairy products like cheese, where it binds easily to casein micelles [61]. It occurs naturally in milk [15] and acts by breaking the β-1,4 bonds between N-acetyl-d-glucosamine and N-acetylmuramic acid residues in the peptidoglycans present in bacterial cell walls [18]. It is especially effective against Gram-positive bacteria. Additionally, it has been found to have antioxidant properties by reducing the formation of reactive oxygen species (ROS) [62].

Cosentino et al. [63] conducted a study on lysozyme in milk. They reported that it has antimicrobial activity against *Bacillus megaterium*, *Bacillus mojavensis*, *Clavibacter michiganensis*, *Clostridium tyrobutyricum*, *Xanthomonas campestris*, and *E. coli*. This activity is similar to synthetic antibiotics and is unaffected by heat treatment. The inhibitory action of lysozyme was also studied by D’ Incecco et al. [64] in 16 hard cheeses made with raw milk. The study demonstrated that cheeses with lysozyme had a lower cultivable microbial count and more DNA from lysed bacterial cells. Furthermore, lysozyme caused the inhibition of *Lactobacillus delbrueckii* and *Lactobacillus fermentum* at the end of 9 and 16 months, respectively.

Ozturkoglu-Budak et al. [65] conducted a study on donkey milk and extracted lysozyme and lactoferrin using FPLC. They applied this extraction to Kashar cheese and found that adding these antimicrobial proteins resulted in cheeses with lower microbial loads. These proteins also helped maintain titratable acidity and increased dry matter content and hardness. They resulted in higher scores for sensory attributes such as appearance, texture, and flavor throughout maturation.

Mehyar et al. [66] conducted a study on halloumi cheese to investigate the effects of chitosan and lysozyme coatings on its microbial quality, shelf life, and sensory properties. The cheese was stored at 3 °C and 25 °C in 5% and 10% w/w NaCl brines. The results showed that lysozyme coatings effectively reduced LAB, psychrotrophic bacteria, anaerobes, molds, and yeasts in cheese salted in a saline solution of up to 15% NaCl. This reduction did not affect the cheese’s sensory properties. In another study, Wang et al. [16] concluded that films with immobilized lysozyme exhibited excellent storage stability and resistance to pH and temperature. The films also effectively reduced *S. aureus* in milk at 4 °C and 25 °C. Furthermore, in a recent study by Awad et al. [17], hydrolyzed peptides from egg white lysozyme showed better antioxidant, sensory, and microbiological effects in yogurt compared to native lysozyme at the same concentration for 28 days of storage.

In addition to reducing bacteria, lysozyme improves sensory characteristics [65,66], particularly texture and storage stability. This may be due to its ability to bind to casein micelles [61]. Furthermore, many studies have evaluated its use in packaging. Finally, hydrolyzed lysozyme appears to favor the formation of peptides with bioactive properties, increasing the functionality of dairy products and presenting greater antimicrobial activity [17].

### 3.2. Lactoferrin

Lactoferrin is a glycoprotein found in mammary gland secretions (comprising 10–20% of total milk proteins) and other exocrine fluids [67]. Its bacteriostatic and bactericidal activity is related to its ability to bind to iron, a substrate for several pathogens such as *Salmonella* spp. and *L. monocytogenes* [68]. Due to its preservative effect and high nutritional value, it has been widely used in dairy products, especially in cheese processing [69,70].

Caputo et al. [71] carried out a study to test the effectiveness of pepsin-hydrolyzed bovine lactoferrin (LFH) as a biopreservative against pseudomonas psychrotrophic pigmenting bacteria, which cause spoilage and blue discoloration of mozzarella cheese. The authors reported that no changes were observed in the casein and pigmentation profiles of the cheese samples inoculated with LFH during 14 days of refrigeration and that this antimicrobial reduced the growth of *Pseudomonas fluorescens* by an average of 2.8 log cfu/g after the storage period. The pigment leucoindigoidin was only detected in cheese samples without LFH [71]. Also, Biernbaum et al. [72] tested the minimum inhibitory concentration of lactoferrin in raw milk against *Salmonella enterica* and *E. coli* O157:H7. The study found that concentrations equal to or greater than 14.06 mg/mL and 112.5 mg/mL of lactoferrin significantly reduced the count of *E. coli* O157:H7 and *S. enterica*, respectively.

A recent study conducted by Adnan et al. [73] aimed to determine the effects of different dosages of lactoferrin (5, 10, 15, 20, and 0 mg/100 g) on the microbiological, proximate, antioxidant, and sensory properties of cheddar cheese. The study revealed that lactoferrin significantly reduced the total viable bacteria count in supplements of 15 and 20 mg/100 g after 45 days of maturation. Additionally, all dosages increased the cheese’s antioxidant capacity without affecting its fatty acid content, proximate composition, color, flavor, and texture scores.

It is worth noting that the compound’s effectiveness in killing bacteria in cheeses can also be influenced by microbial resistance and various factors such as salt concentration, pH levels, moisture, and product storage temperature [74]. For instance, a study by Hassan et al. [75] investigated lactoferrin treatment’s impact on *E. coli* and *S. aureus* strains found in Kareish, Domiati, and Tallaga cheeses. The results indicated that all lactoferrin treatments affected the survival of *E. coli* in Kareish and Domiati cheeses. However, only 20% of the compound reduced *S. aureus* counts in Kareish cheese, and neither concentration significantly inhibited both pathogens in Tallaga cheese.

Although lactoferrin is effective against microorganisms, some heat treatments can reduce its efficacy [76]. Goulding et al. [76] found that temperatures ranging from 72 to 95 °C can permanently alter the physical and chemical properties of bovine lactoferrin, making it unsuitable for use in dairy products that undergo heat treatments at higher temperatures, such as baked cheese production at 72 °C.

### 3.3. Chitosan

Among the most abundant and well-known natural biopolymers, chitosan, extracted from insects, fungi, and crustaceans, stands out as an amino polysaccharide with excellent preservative properties. The food, medical, and pharmaceutical industries commonly use its purified form and derivative products [77]. Chitosan is estimated to have had a global market share of USD 10.88 billion in 2022 and is predicted to increase to USD 47.06 billion by 2030 [78]. As it is non-toxic, odorless, biodegradable, and easily absorbed by the body, its application is extensive in dairy products, mainly microencapsulated or as coating films [79].

Alfaifi, Alkabli, and Elshaarawy [80] have developed a preservative using water-soluble chitosan (WSC) and 2-azido propanoic acid (APA). They have reported that the minimum inhibitory concentration (MIC) of WSC-APA completely suppressed the growth of *E. coli* O157:H7 and *S. aureus* in raw milk samples. The refrigerated milk samples were kept for 20 and 24 h, respectively. Additionally, concentrations of 0.25 mg/mL of these associated compounds were sufficient to eliminate *Staphylococcus* spp. after six days of storage. Moreover, there was a 99.7% reduction in coliforms after 10 days.

Chitosan is a substance that can increase the shelf life of yogurts, improve their consistency, and reduce syneresis. According to a study by Zedan, Hosseini, and Mohammadi [81], adding high molecular weight chitosan (at concentrations of 2%, 4%, or 6%) to yogurt with Artemisia oil dracunculus can have positive effects. Samples containing 6% chitosan and 20% essential oils had a higher pH, lower acidity, and syneresis and a proportional decrease in the count of total bacteria and yeasts as the chitosan concentration increased. However, in the sensory evaluation, samples with only 2% chitosan received the highest overall score, indicating that higher concentrations may negatively affect consumer acceptance.

Fungi often contaminate cheese surfaces. However, it has been reported that chitosan incorporated into coating films can prevent such contamination. Dıblan et al. [82] conducted a study comparing different active films on the stability and microbial quality of kaşar cheese, concluding that chitosan films were more effective in controlling yeasts and fungi than films of potassium sorbate, nisin, and silver-substituted zeolite. In another study, Dong et al. [83] combined a cell-free supernatant (CFS) of *Lacticaseibacillus paracasei* ALAC-4 with a chitosan matrix in the active packaging of Mongolian cheese. This combination significantly inhibited molds and yeasts, particularly *Candida albicans*, for 15 days during storage at 4 °C. The chitosan matrix also provided excellent mechanical properties. Despite its favorable results, chitosan is often used with other antimicrobial agents, which limits the verification of its effectiveness against some microorganisms.

## 4. Antimicrobials from Plant Sources

In recent years, there has been a growing interest in exploring plant-derived preservatives in the dairy sector. Researchers have been particularly intrigued by the potential of plant extracts from vegetable by-products, herbs, and spices. This is because certain plants are known to thrive in harsh environments and produce compounds that possess insecticidal, fungicidal, antibacterial, and antiviral properties [84]. Based on their main properties, these compounds can be classified into different chemical groups, such as terpenes, phenylpropenes, allicin, and isothiocyanates [85]. The antimicrobial properties of these compounds are well documented, and they have been shown to interact with the bacterial cell membrane in a way that disrupts its structure [86]. This makes them highly effective in preserving food, particularly dairy products, which are prone to spoilage due to their high nutrient content. Additionally, plant-derived preservatives have a high antioxidant capacity, which makes them useful in preventing oxidative damage to food. The potential of plant-derived preservatives in the dairy industry is very promising. These compounds have been shown to possess many desirable properties, making them an attractive alternative to synthetic preservatives [26]. If harnessed correctly, they could play a significant role in food preservation, ensuring that dairy products remain fresh and safe for consumption.

### 4.1. Plant extracts

Plant extracts can be either liquid or solid. They can be extracted through various methods such as maceration, digestion, infusion, and decoction or emerging techniques such as Accelerated Solvent Extraction (ASE) and Ultrasound-Assisted Extraction [87]. The literature has widely reported the antimicrobial activity of these compounds in dairy products [19,20,21,22]. Moreira et al. [19] investigated the effects of decreasing the concentration of sodium chloride on the microbiological quality of fresh goat’s cheese. They achieved this by adding pequi extract (*Caryocar brasiliense*) to pasteurized milk (CM) and cheese mass (CS), and by testing it under immersion (CIE). The results showed a significant decrease in LAB counts in CS and CIE samples after 21 days of storage. No presence and count of Enterobacteriaceae, *Staphylococcus* spp., and *E. coli* were observed in any samples during storage [19].

In a study by Wanniatie et al. [20], the effect of adding red ginger extract to goat’s milk yogurt was investigated. The results showed that the total bacterial count decreased significantly after adding 2% extract, but 4% extract increased the LAB count. In another study by Kamel et al. [21], carrot powder was incorporated into soft buffalo cheese with probiotics. The total bacterial count decreased from 7.5 to 7.3 log CFU/g in the product added with 0.6% of the compound. However, the LAB and *Bifidobacterium longum* count increased at the end of 28 days for dosages of 0.4% and 0.6%. In the sensory evaluation, samples with 0.6% carrot powder obtained the lowest flavor attribute score on the first storage day. However, no significant differences were observed in the flavor, texture, color, and appearance scores among all of the samples at the end of the storage period.

Ávila Arribas et al. [22] also obtained satisfactory results when comparing the inhibitory effect of oregano plant extract (*Origanum vulgare*), savory (*Satureja montana*), hyssop (*Hissopus officinalis*), and tarragon (*Artemisia dracunculus*) on the growth of different strains of *Clostridium* spp. in cheeses. The authors reported that all Clostridium strains were sensitive to EEs in at least one of the concentrations tested and that EEs from hyssop, lavender, and tarragon had lower MICs (<40 μL/mL). EEs from savory, lavender, and tarragon also delayed the appearance of vegetative cells of *Clostridium* spp. and delayed stewing for two weeks without harming the sensorial characteristics of the cheese.

Shehata et al. [88] evaluated the antimicrobial effect of taro leaf extract (TLE) at concentrations of 250 and 500 mg/L. The researchers found that this compound exhibited excellent activities against various microorganisms, including *E. coli* BA 12296, *Salmonella Senftenberg* ATCC 8400, *Fusarium oxysporum* ITEM 12591, and *S. aureus*, with inhibition zones of 19.3 ± 1.02 mm, 18.53 ± 0.75 mm, 12.93 ± 1.17 mm, and 11.33 ± 0.84 mm, respectively. Additionally, the extract enhanced the antioxidant potential, the viability of *L. paracasei*, and the concentration of polyphenols in the drinks. These findings demonstrated good acceptability by consumers.

Plant extracts have become increasingly important in processing functional foods and are used as alternatives to synthetic antimicrobials. Certain extracts’ effectiveness against sporulating bacteria that cause late puffing in matured cheeses is a significant advantage. This defect is challenging to control and can result in significant losses for the food industry. Additionally, when the extracts are derived from industrial waste, they have greater environmental appeal and add value to the products.

### 4.2. Essential Oils

Essential oils (EOs) are volatile hydrophobic liquids that various plants produce to protect themselves against bacterial, viral, and fungal infections [89]. These oils are highly effective in inhibiting the growth of Gram-negative bacteria due to their hydrophobic nature. This property enables the oils to easily penetrate the lipopolysaccharide barrier of the outer membrane of these microorganisms. Once inside the bacterial cells, they disrupt the transportation of molecules, leading to the eventual death of the cells [90]. It is important to note that the efficacy of EOs varies between different bacterial strains, and their minimum inhibitory concentrations (MICs) should be considered. The MIC is the lowest concentration of EOs needed to inhibit the growth of a particular bacterial strain. The MIC differs from one bacterial species to another and is also influenced by the source of the EO. Therefore, it is essential to assess the MIC of EOs to determine their optimal concentration for specific applications [23].

Several EOs have already been studied in dairy products, such as thyme [91]; cinnamon [92]; oregano [93,94]; pink pepper [95]; lemon [96,97]; perilla leaf [98]; rosemary [99]; tangerine and orange [96]; mastic [100]; and curry and cloves [101], demonstrating high preservative action, in addition to increasing shelf life and improving physicochemical and sensory characteristics, mainly due to the volatile compounds. Jemaa et al. [91] compared the effect of thyme EO (Thymus capitatos) and its nanoemulsion on the quality of raw milk contaminated by *S. aureus*, demonstrating greater efficiency of the nanoemulsion in inhibiting the pathogen. However, the milk with the EO had greater antioxidant capacity and lower protein degradation. Furthermore, there were no statistical differences in the acidity content and peroxide inhibition between both treatments, demonstrating that thyme EO in solution or nanoencapsulated can improve milk quality and extend its shelf life.

The antimicrobial efficacy of cinnamon essential oil (EO) in milk was examined by Abbes et al. [92]. The results showed that *Salmonella* was completely inactivated at 3 μg mL^−1^. Similarly, Hao et al. [94] investigated the antimicrobial properties of oregano EO, rich in carvacrol, against *E. coli* and *S. aureus* in milk. They used nuclear magnetic resonance (NMR) to study the mechanism of action and observed bacteriostatic and bactericidal effects, which were associated with changes in the cellular morphology of both microorganisms. In a previous study, Campos et al. [93] analyzed the inhibitory effect of oregano EO on filamentous fungi (*Aspergillus flavus*, *Fusarium oxysporum*, and *Penicillium citrinum*) as well as *E. coli* and *S. aureus* during the ripening of cheese. Oregano EO at a concentration of 0.02% w/w inhibited the growth of the tested strains over the 30-day maturation period. It reduced 7 logs (CFU/g) of *S. aureus* in the first hour of maturation and suppressed all viable cells of *E. coli* after three days of maturation. There were no changes in the pH and moisture of the cheese.

*L. monocytogenes* is a difficult pathogen to control in cheese production. A recent study by Dannenberg et al. [95] evaluated the efficacy of pink pepper essential oil (EO) as an antioxidant and antimicrobial agent in Minas Frescal cheese. The study also tested the oil against 18 bacteria in vitro. The study showed a reduction of 1.3 log CFU/g in *L. monocytogenes* within 30 days, along with the inhibitory activity against seven spoilage bacteria, six pathogenic bacteria, and three bacteria with technological applications. In addition, there was a reduction in free radical DPPH. Another study by Fancello et al. [97] evaluated the effect of lemon EO (*Citrus limon var pomia*) on ricotta salata cheese stored at 5 °C. The study showed that the EO had bactericidal effects on *L. monocytogenes* DSMZ and bacteriostatic effects on a mixture of *L. monocytogenes* strains. The chemical analysis of the liquid phase of this EO revealed that its main active compounds were linalyl acetate, limonene, and two isomers of citral.

Numerous studies have shown that essential oils (EOs) can enhance the quality of yogurts’ microbiological, physical, chemical, and sensory characteristics [98,99,102,103]. For example, He et al. [98] investigated the impact of perilla leaf EO on the production of volatile compounds and the microbiological quality of yogurt. They found that adding 0.04% of this EO resulted in a longer shelf life, the production of 69 volatile compounds (mainly limonenes), an increased concentration of terpenic substances, and greater sensory acceptance compared to the control yogurt. Similarly, Kamel et al. [99] examined the properties of rosemary EO as an alternative to synthetic preservatives in yogurt. They reported that rosemary EO exhibited antimicrobial activity against microorganisms such as *E. coli*, *S. aureus*, *Salmonella marcescens*, total coliforms, yeasts (*Candida albicans*), and fungi (*Aspergillus flavus*). Moreover, adding rosemary EO enhanced the viability of LAB and improved the overall acceptance of the yogurt.

Ice creams also represent a dairy matrix favorable to the addition of essential oils, as they are among the most consumed dairy products and are highly accepted among different age groups [104]. For example, EOs obtained from fruit residues from industrial processes are sustainable alternatives for incorporating natural antimicrobials into edible ice creams [96]. Tomar and Akarca [96] evaluated the microbiological, physical, chemical, and sensory properties of ice creams produced with EOs from lemon, tangerine, and orange peels at different concentrations (0.1%, 0.3%, and 0.5%). In conclusion, the authors did not identify the presence of total coliforms, *Salmonella* spp., *S. aureus*, *E. coli*, and *L. monocytogenes* in the samples. Furthermore, ice cream added with 0.5% EO and orange peel stood out, having the lowest counts of aerobic mesophilic bacteria (3.80 log CFU/g), yeasts and fungi (3.71 log CFU/g), psychrophilic bacteria (4.01 log CFU/g), and *Pseudomonas* spp. (2.04 log CFU/g).

Mitropoulou et al. [100] researched ice cream to determine the minimum inhibitory concentration of microorganisms and the main active compounds of mastic EO (*Pistacia lentiscus*) by GC/MS. The study found that ice cream containing 0.2% (*w*/*w*) of EO gradually decreased the counts of *E. coli*, *L. monocytogenes*, and *Pseudomonas fragi* during the first seven weeks of storage. On average, the count was reduced by 3 log CFU/g compared to the control samples (*p* < 0.05). The primary constituents of the EO that contributed to this reduction were a-pinene (67.7%), myrcene (18.8%), and β-pinene (3.0%).

The use of EOs in dairy-based desserts has also been reported. Badola et al. [101] added EOs from curry leaves (0.05–0.15 ppm) and cloves (0.15–0.25 ppm) and evaluated the microbiological, antioxidant, physicochemical, and sensory characteristics. In addition to the greater antioxidant activity and greater presence of total phenolics, there was a reduction in the count of total bacteria, molds, and yeasts.

Although the numerous technological and antimicrobial properties of EOs are reported, their incorporation into the formulation of dairy products can also be limited by several factors [105]. For example, volatile organic compounds can quickly decompose in adverse environmental conditions, leading to a loss of flavor, aroma, and antioxidant and antimicrobial efficacy [106]. Furthermore, antimicrobial phytochemicals in high concentrations can present toxicity and allergenic potential in sensitive individuals [107], in addition to possible interactions with food components, damaging their quality [108].

Although essential oils (EOs) have been shown to have antimicrobial properties, their use in certain dairy products should be limited. This is because they can negatively affect the product’s sensory qualities [100,101]. In some cases, the undesirable flavor may be due to the presence of certain phytochemicals such as 3-carene, caryophyllene [109], and eugenol [110]. This issue is particularly noticeable in dairy products with a high-fat content.

In conclusion, the minimum inhibitory concentrations (MICs) of different EOs for different bacterial strains should be considered. Several EOs have been studied in dairy products, showing high preservative action, increased shelf life, and improved physicochemical and sensory characteristics. EOs have also been shown to enhance quality, mainly by improving yogurt’s microbiological, physicochemical, and sensory characteristics.

## 5. Encapsulation of Natural Antimicrobials

As previously mentioned, the effectiveness of antimicrobial compounds can be reduced by factors such as temperature, moisture, exposure to light, and undesirable interactions with dairy matrix constituents [70]. The encapsulation of these compounds is a viable technique for the protection and controlled release of natural antimicrobials during the production or storage of products [9]. For instance, one encapsulation method involves using liposomes, which are small spherical vesicles with a lipid bilayer. These liposomes can encapsulate the antimicrobial compounds, protecting them from the surrounding environment. Another method is microencapsulation, where the antimicrobial compounds are encapsulated in a polymer matrix. This technique temporarily retains an active substance within a shell-forming material, releasing it under specific conditions such as water activity, time, temperature, or pH [111]. In summary, encapsulation can increase the stability of natural antimicrobial compounds from different sources in dairy matrices, minimizing the risks of inactivation and loss of efficacy over time.

Various applications of natural antimicrobial compounds encapsulated in dairy products have been explored in the literature, including the use of polymeric nanocapsules [112], nanofibers [102], agar microcapsules [113], soy protein isolates [114], and chitooligosaccharides [115]. These studies have shown promising results, particularly in enhancing these products’ quality, safety, and biological activity. However, selecting an appropriate encapsulation system must consider the interactions between the wall material with the antimicrobial compounds and the components of the food matrix to ensure better chemical stability of the substance and its delivery to the target [116]. For example, polymeric nanoparticles loaded with *Baccharis dracunculifolia* DC essential oil proved effective when tested in BHI broth against *S. aureus*, *B. cereus*, *L. monocytogenes*, and *S. enteritidis*; however, when evaluated in milk against *L. monocytogenes*, reduced activity was observed, possibly due to the interaction with milk components [112]. The production of nanoparticles from *Nepeta crispa* oil has also been effective against Gram-negative bacteria such as *E. coli* and *S. aureus*, in addition to improving functional and sensory characteristics in yogurt [102]. Furthermore, it is important to mention that the encapsulation of natural compounds also increases the biological activity of dairy products, in addition to their benefits against microorganisms. For example, encapsulating essential oils with agar effectively protects their antioxidant activity during digestion [102,113]. It is important to note that more research is needed, especially considering that the majority of studies on dairy matrices are focused on milk [117,118], yogurts [102,115], and films for cheeses [48,119].

## 6. Methodology

To gather relevant data for a review on natural antimicrobials in dairy products, a comprehensive literature search was conducted using three prominent academic databases: Science Direct, PubMed, and Scopus. The initial search used “natural antimicrobials” and “dairy products”. Subsequently, additional search terms, including “bacteriocins,” “antimicrobial enzymes,” “plant extracts,” “essential oils,” “milk,” “fermented milk,” “yogurt,” “cheese,” and “dairy drink,” were also used to refine the search. Priority was given to articles published within the last five years to ensure that the most recent research was included. However, specific older articles which contained significant discussions relevant to the review were also included in the search. In addition, Table 1 summarizes the studies that used antimicrobials in dairy products and their main findings.

## 7. Conclusions

Dairy products are commonly exposed to microorganisms that can spoil the food and cause harm to human health. To counter this, researchers have investigated different sources and applications of natural antimicrobials that are effective against specific microorganisms found in dairy products. These antimicrobials can originate from microbes, animals, and plants. For instance, some natural antimicrobials such as lysozyme positively affect dairy products, particularly the sensory and textural aspects. However, inconsistency exists in the literature regarding some factors, such as the ideal intrinsic conditions for the action of certain antimicrobials. Additionally, standardization is crucial to accurately interpret the results of studies, as the metabolism of the microorganism producing the antimicrobial can affect the results. Using natural antimicrobials in packaging and active films is a growing trend that can benefit dairy products during storage. However, natural antimicrobials, such as essential oils, can produce off-flavors in high-fat dairy products. Further studies are necessary to develop natural antimicrobials and validate their use in dairy products to better understand their effects and interactions with the food matrix and active packaging and their impact on untested microorganisms.

## Figures and Tables

**Table 1:** Main studies on natural antimicrobials in dairy products.

Natural Antimicrobial	Dairy Product	Dosage	Main Results and Target Microorganism	References
Nisin	Minas Frescal cheese	500 IU·mL^−1^	It increased the lag phase of *S. aureus.* It resulted in a decrease in *S. aureus* counts in cheese dough and whey.	[34]
Nisin	Dairy beverage	3 MICs	Associated with lactose laurate, it inhibited the growth of *S. aureus* for 10 days, stabilizing the pH and relative viscosity.	[36]
Natamicin	Mozarella	0.25, 0.5, and 1 mg/dm^2^	The dosage of 1 mg/dm^2^ of natamycin combined with a hydroxyethylcellulose film and pulverized reduced 5.28 Log CFU/g and 4.19 Log CFU/g (99.9%) of the fungal population of *Penicillium* spp. It increased the shelf life.	[46]
Natamicin	Dairy beverage	1% pp/p	Films incorporated with 1% natamycin and treated with UV rays for 6 min provided maximum antiyeast activity against *Rhodotorula mucilaginosa* and *Candida parapsilosis*.	[53]
Reuterin (*Lactobacillus reuteri*)	Fresh cheese	6 log^10^ cfu of Lb. reuteri	Adding *L. reuteri* significantly decreased (*p* > 0.05) the *E. coli* O157:H7 population in fresh cheeses after 28 days in 10% or 15% brine at 10 °C or 25 °C.	[58]
Reuterin	Yogurt	1.38 mM and 6.9 mM	Reuterin at a concentration of 1.38 mM showed a fungistatic effect, and at a concentration of 6.9 mM, it showed a fungicidal effect against a representative panel of contaminating fungi in dairy products.	[54]
Lisozyme	Milk	1.09 mg/L	It inhibited the growth of *Bacillus megaterium*, *Bacillus mojavensis*, *Clavibacter michiganensis*, *Clostridium tyrobutyricum*, *Xanthomonas campestris*, and *E. coli*, with an action similar to ampicillin and kanamycin. The exception was *Bacillus mojavensis*, which showed resistance to lysozyme in milk samples subjected to heat treatment.	[63]
Lysozyme-hydrolyzed peptides	Yogurt	0.4%	Inhibition of molds and yeasts during 28 days of storage. It resulted in an increased antioxidant capacity. Higher scores for color, appearance, taste, and overall acceptance.	[17]
Lactoferrin	Milk	14.06 mg/mL and 112.5 mg/mL	Significant decrease (*p* ≤ 0.05) in the count of *E. coli* O157:H7 and *Salmonella enterica* at levels equal to or greater than 14.06 mg/mL and 112.5 mg/mL of lactoferrin, respectively.	[72]
Lactoferrin	Cheddar	5, 10, 15, and 20 mg/100 g	After 45 days, viable bacterial counts significantly decreased at doses of 15 and 20 mg/100 g. There was a 22% decrease in the total viable bacterial count and a 72% increase in the antioxidant capacity at a dosage of 20 mg/100 g. All dosages significantly increased the antioxidant capacity, but there was no change in the proximate and fatty acid composition or in the cheese’s color, flavor, and texture scores.	[73]
Conjugate of chitosan and azidopropanoic acid	Milk	0.25 mg/mL	It resulted in the total inhibition of the *E. coli* O157:H7 and *S. aureus* population in raw milk refrigerated at 4 °C after 20 and 24 h, respectively. No species of *S. aureaus* were detected in milk after 6 days of storage. In total, a 99.7% reduction in coliform counts after 10 days of storage.	[80]
Chitosan	Kasar cheese	2% p/p	Incorporated into the active film, it promoted the total inhibition of yeasts and fungi in the cheese after 20 days of storage at 15 °C, a 1.5 log reduction in *S. aureus* counts, and an increase in titratable acidity.	[82]
Pequi extract (*Caryocar brasiliense*)	Caprin fresh cheese	6.25 mL/L	Cheeses with the extract added to the dough or those immersed in the extract exhibited a significant decrease (*p* < 0.05) in LAB counts after 21 days of storage. Samples added from the extract showed lower luminosity (*p* < 0.05). The cheese added with pequi extract to the dough showed greater hardness (*p* < 0.05).	[19]
Red ginger extract	Goat’s milk yogurt	1%, 2%, 3%, and 4% p/p	There was a decrease in the total viable bacterial count after adding 2% of the extract. The addition of 2% extract reduced the viscosity, density, and LAB count, but the addition of 4% extract increased the LAB count.	[20]
Oregano OE	Cured cheese	0.02% p/p	It resulted in the inhibition of the growth of *Aspergillus flavus*, *Fusarium oxysporum*, *Penicillium citrinum*, *E. coli*, and *S. aureus* during 30 days of maturation. It resulted in a reduction of seven logs in the *S. aureus* population in the first hour of ripening and the inhibition of all viable *E. coli* cells after three days of ripening, without changes in the pH and moisture of the cheeses.	[93]
Aroeira OE	Ice cream	0.2% p/p	It resuled in a decrease of approximately 3 log cfu/g in the counts of *E. coli*, *L. monocytogenes*, and *P. fragi* until the seventh week of storage. There was a low sensory acceptance.	[100]

## Data Availability

Not applicable.
